# Dose-Response Relationship between Endurance Training Prescription Variables and Increases in Aerobic Performance of Healthy and Unhealthy Middle and Very Old Individuals Aged 70 Years and Older: A Systematic Review and Meta-Analysis of Randomized Controlled Trials

**DOI:** 10.3390/life11020121

**Published:** 2021-02-05

**Authors:** Sarah Cheour, Chouaib Cheour, Nicola Luigi Bragazzi, Liye Zou, Armin H. Paravlic, Maamer Slimani, Foued Cheour

**Affiliations:** 1 High Institute of Sport and Education of Sfax, Sfax 3000, Tunisia; sarah.cheour@yahoo.com (S.C.); chouaib.cheour@yahoo.com (C.C.); 2 Laboratory for Industrial and Applied Mathematics (LIAM), Department of Mathematics and Statistics, York University, Toronto, ON M3J 1P3, Canada; robertobragazzi@gmail.com; 3 College of Psychology and Sociology, Exercise and Mental Health Laboratory, Shenzhen University, Shenzhen 518060, China; liyezou123@gmail.com; 4 Institute of Kinesiology Research, Science and Research Centre, SI-6000 Koper, Slovenia; armin.paravlic@hotmail.com; 5 Faculty of Sport, University of Ljubljana, 1000 Ljubljana, Slovenia; 6 Department of Neuroscience, Rehabilitation, Ophthalmology, Genetics, Maternal and Child Health (DINOGMI), Section of Psychiatry, Genoa University, 16126 Genoa, Italy; 7 School of Public Health, Department of Health Sciences (DISSAL), Genoa University, 16126 Genoa, Italy; 8 High Institute of Applied Biology of Médenine, Medenine 4119, Tunisia; fouedcheour@yahoo.fr

**Keywords:** exercise, elderly, physical endurance, physical fitness, physical activity training prescriptions, systematic review and meta-analysis, dose–response relationship

## Abstract

Background: The objectives of this systematic review and meta-analysis were to quantify the effectiveness of endurance training (ET) on aerobic performance (i.e., peak oxygen uptake (VO_2peak_)) in healthy and unhealthy middle and very old adults aged 70 years and older, and to provide dose–response relationships of training prescription variables (in terms of frequency, and volume). Methods: Several scholarly databases (i.e., PubMed/MEDLINE, SpringerLink, ScienceDirect Journals, and Taylor & Francis Online—Journals) were searched, identifying randomized controlled studies that investigated the effectiveness of ET on VO_2peak_ in older adults. Standardized mean differences (SMD) were calculated. Results: In terms of changes differences between experimental and control group, ET produced significant large effects on VO_2peak_ performance (SMD = 2.64 (95%CI 0.97–4.31)). The moderator analysis revealed that “health status” variable moderated ET effect onVO_2peak_ performance. More specifically, ET produced larger SMD magnitudes on VO_2peak_ performance in healthy compared with unhealthy individuals. With regard to the dose–response relationships, findings from the meta-regression showed that none of the included training prescription variables predicted ET effects on VO_2peak_ performance. Conclusions: ET is an effective mean for improving aerobic performance in healthy older adults when compared with their unhealthy counterparts.

## 1. Introduction

Ageing is a complex, multi-factorial psycho-biological process [1], which affects different organs and body systems, including heart, muscles, and lung [2]. In more detail, cardiac aging is characterized by decrease in left ventricular diastolic function [3] as well as by senescence of cardiomyocytes and cardiac stromal cells [4] and decreased cardiovagal baroreflex sensitivity [5], potentially resulting in heart failure and atrial fibrillation. Muscle size is reduced, and architecture altered in old age [6], with the number of motor units and muscle fibers being significantly decreased [7]. As a result, muscle strength and power tend to decline by 3–5% per year [8]. Taken together, sarcopenia and dynapenia lead to an increased risk of falls, frailty, disability and, ultimately, to death [9,10].

Physical activity and structured exercise can offset these ageing-related decreases in human functions. In particular, enhancing cardiovascular fitness can result in substantial health benefits, positively impacting independence and health-related quality of life [11]. Of note, it is well-known that endurance training (ET) can significantly improve peak oxygen consumption by approximately 10–15% in older adults [12,13]. For instance, improvement in peak oxygen uptake (VO_2peak_) is influenced by training volume and frequency in young individuals [12,13]. However, there is a dearth of information regarding the impact of different training prescription variables (in terms of type of training and session duration, and weekly training frequency) on VO_2peak_performance among healthy and unhealthy elderly [14,15]. In this sense, determining the most effective dose that leads to enhancements in aerobic capacity has become a relevant topic in the scientific literature [16].

Therefore, the aims of this systematic review and meta-analysis of available randomized controlled trials (RCTs) will be to quantify the effectiveness of ET on VO_2peak_ performance in healthy and unhealthy older adults (middle and very old adults, aged 70 years and older) and to provide dose–response relationships of various training prescription variables (frequency and volume). This can be extremely helpful for clinicians and allied health professionals in designing ad hoc programs and interventions for old and frail individuals. It can be expected that, by specifically addressing the effects of training prescription variables, the present systematic review and meta-analysis will enhance current knowledge, especially about the dose–response relationship.

## 2. Materials and Methods

### 2.1. Literature Search Strategy

**Figure 1:**
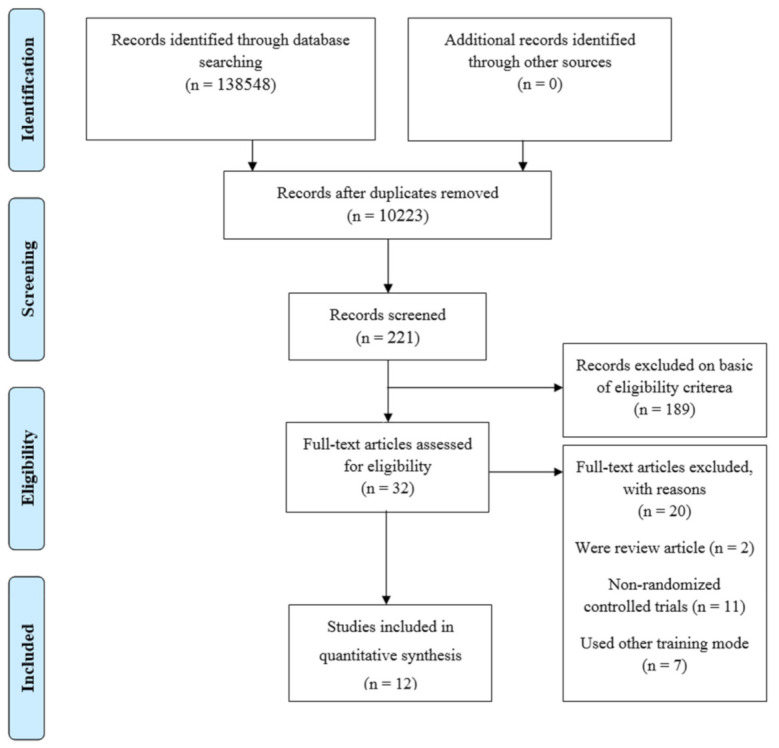
“Preferred Reporting Items for Systematic Reviews and Meta-analysis” (PRISMA) flow-chart, n: number.

The “Preferred Reporting Items for Systematic Reviews and Meta-Analysis” (PRISMA) guidelines were used to conduct this systematic review and meta-analysis (Figure 1, [17]). RCTs that investigated the effects of ET on VO_2peak_ in healthy and unhealthy older adults were obtained through systematic manual and electronic searches (up to 15 August 2018) in electronic databases (i.e., PubMed/MEDLINE, ScienceDirect Journals, SpringerLink, and Taylor & Francis Online—Journals). Electronic databases were searched using the following search syntax with keywords and/or MeSH terms, where appropriate: “endurance training” OR “aerobic training” AND “older” OR “senior” OR “elderly” AND “VO_2peak_” OR “aerobic performance”. Moreover, relevant references were manually inspected from published articles to increase the chance of getting potentially relevant studies. Google Scholar was used to retrieve and capture potentially related investigations, reviewing their full-text version.

### 2.2. Risk of Bias Assessment

According to the Cochrane Collaboration guidelines [18], the methodological quality and risk of bias were assessed independently by two authors via visual interpretation of funnel plots.

### 2.3. Inclusion and Exclusion Criteria

Studies were selected according to the Population/Intervention/Comparison/Outcome(s)/Study design (PICOS) criteria:

1. Population: studies involving healthy and unhealthy (i.e., patients with heart failure and preserved ejection fraction, individuals with ischemic heart disease, hypertension, musculoskeletal problem, respiratory disease, diabetes, overweight or obese) middle and very old adults (aged 70 years and older).
2. Intervention or exposure:
3. Studies investigating the effects of ET on VO_2peak_ in older adults;
4. Comparator: Studies comparing an experimental group with a control group;
5. Outcome(s):
6. VO_2peak_ (L/min or mL/kg/min);
7. Training prescription variables: type of training, training frequency (sessions/week), and training duration (weeks);
8. Study design: RCTs studies;
9. Studies were excluded if:
  (i) Reviews, opinion papers and commentaries, interviews, editorials, posters, conference papers, letters to the editor, book chapters, and books.
  (ii) Articles with insufficient data.

### 2.4. Coding of Studies

A structured form was used to extract data by two authors. According to some meta-analyses [19,20] and the included studies, training prescription variables were grouped into the following areas: (i) characteristics of participants: health status (healthy vs. unhealthy) and gender (male vs. female vs. combined) and (ii) training prescription variables including training duration in weeks (<13 vs. ≥13 weeks), weekly training frequency (3 vs. 4–5 sessions per week), and session duration (20–30 min vs. 31–45 min vs. 46–65 min).

Since the values of the training prescription variables (namely, training duration in weeks, weekly training frequency, and session duration) varied among the studies, cut-off/threshold values were identified based on median values and previous meta-analysis [19] to allow comparing the various studies.

### 2.5. Data Extraction

The characteristics (i.e., training prescription variables, and performance outcomes) of each study were extracted in anad hoc Excel template/spreadsheet.

### 2.6. Statistical Analyses

In this systematic review and meta-analysis, a standardized documentation form was used to extract relevant data from the identified studies in terms of changes differences between experimental and control group. The main outcome measure was the standardized mean difference (SMD). Some studies comprised multiple experimental and control groups, which caused the redundancy and the dependency of the same group in more than one contrast. To avoid the dependency, several solutions have been proposed by Assink and Wibbelink [21]: analyzing the outcomes as if they were independent, averaging the dependent outcomes into a single effect size, selecting only one outcome for each study, and multi-level meta-analysis. So far, the first two solutions might bias the results and decrease the power of the analysis. For instance, we chose a multi-level meta-analysis model, considering three different sources of variance: the participants at level one, the outcomes at level two, and the studies at level three.

For this reason, R programme (Version 4.0.3, [22]), RStudio (Version 1.4.1103, [23]) together with Metafor package [24] was used to conduct the analyses. We followed all procedures as proposed by Assink and Wibbelink [21]. Briefly, we used the rma.mv function of the “Metafor” package and set the t distribution (T.DIST) parameter as TRUE. Therefore, we based the test statistics and confidence intervals on the T.DIST, applied the Knapp and Hartung [25] adjustment, and used the Restricted Maximum Likelihood estimation method (REML) for estimating the parameters. A funnel plot was used to determine potential publication bias by looking at the asymmetry of the graph. In addition, meta-regression analyses (Mixed-Effects Model) by using “Metareg” function in “Meta” package, were computed to determine the possible training prescription variables (e.g., weekly training frequency, training duration, number of exercises, number of repetitions per sets, and number of sets per training) that may have influenced training-related effects. SMD magnitudes were considered as trivial (<0.35), small (0.35–0.80), moderate (0.80–1.50), or large (>1.5) [20]. The significance level was set at *p* < 0.05.

## 3. Results

### 3.1. Literature Search Results

After reviewing the titles and abstracts of 138,548 studies, 221 papers remained for further scrutiny. According to the inclusion and exclusion criteria, full texts of 32 articles were retrieved and assessed. After a careful review of the full texts, 12 articles [26,27,28,29,30,31,32,33,34,35,36,37] met the inclusion criteria and were, as such, selected in this systematic review and meta-analysis. A flow chart of the systematic search process is illustrated in Figure 1. Details of all included studies, totaling a sample of 407 initially recruited subjects (322 considering only controls and cases involved in ET and excluding other types of interventions).

### 3.2. Overall Effects of Endurance Training on Measures of VO_2peak_

Our analyses revealed that ET had a large effect onVO_2peak_ performance in healthy and unhealthy individuals (SMD=2.64 (95%CI 0.97–4.31), t_17_ = 3.33, *p* = 0.004), with significant heterogeneity (Q = 128.41, *p* < 0.001) (Table 1). Further, looking at the distribution of the total variance over the three levels we found that 3.39%, 8.38%, and 88.23% can be attributed to variance at level 1, level 2, and level 3, respectively. Following the 75% rule proposed by Raudenbush et al. [38], suggesting that if less than 75% of the total amount of variance can be attributed to sampling variance at level 1, the heterogeneity can be regarded as substantial. Therefore, we proceeded to examine potential moderators of the effect of the intervention on VO_2peak_ of older adults.

### 3.3. Influence of Different Moderating Variables on Endurance Training Related Effects

The following moderating variables were studied: health status (healthy, unhealthy and both/combined—healthy and unhealthy), gender (male, female and both/combined—male and female), training and session duration, and training frequency.

#### 3.3.1. Health Status

There was a statistically significant effect of the moderator variable “health status” (healthy vs. unhealthy vs. both/combined (healthy and unhealthy)) on VO_2peak_ performance (F_1,15_ = 13.53, *p* < 0.001). ET produced larger effect on VO_2peak_ performance in the combined healthy and unhealthy group compared with healthy and unhealthy individuals (Table 1).

#### 3.3.2. Gender

Our subgroup analyses indicated that ET produced larger SMD magnitudes on VO_2peak_ performance in females (SMD = 3.63, (95%CI 0.11–7.14), t_15_ = 2.18, *p* = 0.04); compared with males (SMD = 2.23, (95%CI −0.31–4.71), t_15_ = 1.84,*p* = 0.085; and the combined group (SMD = 1.38, (95%CI −0.54–3.30), t_15_ = 1.52,*p* = 0.14), while difference between groups where not significant (F_1,15_ = 0.46–3.17, *p* = 0.07–0.63).

### 3.4. Dose–Response Relationships of Endurance Training on VO_2peak_

#### 3.4.1. Findings from the Meta-Regression

Findings from the meta-regression showed that none of the included training prescription variables could predict ET effects on VO_2peak_ performance (training duration: coefficient of estimate (CE) = 0.07, *p* = 0.29; session duration: CE = −0.007, *p*=0.90; and weekly training frequency: CE = 0.01, *p* = 0.73).

#### 3.4.2. Findings from the Univariate Analysis

##### Training and Session Duration and Training Frequency

There were no significant differences between the observed training period (i.e., <13 vs. ≥13 weeks) for measures of VO_2peak_ (F_1,16_ = 0.54, *p* = 0.47), as well as no differences could be detected between the observed session duration (i.e., 20–30 min vs. 31–45 min vs. 46–65 min) for measures of VO_2peak_ (F_1,16_ = 1.01, *p* = 0.37). Similarly, there were no significant differences between the observed weekly training frequencies (i.e., 3 vs. 4–5 session per week) for measures of VO_2peak_ (F_1,16_ = 0.11, *p* = 0.73) (Table 1).

### 3.5. Evaluation of Publication Bias

**Figure 2:**
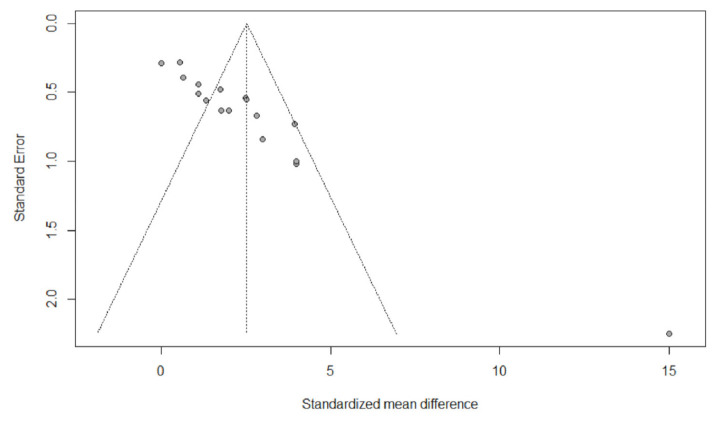
Funnel plot of standard difference in means versus standard error for VO_2peak_; the aggregated standard difference in means is the random-effect mean effect size weighted by degrees of freedom.

Figure 2 shows asymmetric funnel plots which indicates the presence of publication bias in studies assessing the effects of ET on VO_2peak_ performance.

## 4. Discussion

This is the first systematic review and meta-analysis examining ET specific dose–response relationships for VO_2peak_performance according to the training prescription variables in healthy and unhealthy middle and very old individuals (aged 70 years and above). We found that, overall, ET had a large effect on VO_2peak_ performance in healthy and unhealthy individuals. Subgroup analysis demonstrated that healthy individuals reported larger improvements in VO_2peak_ performance than unhealthy individuals.

### 4.1. General Effectiveness of Endurance Training on VO_2peak_

Some systematic reviews and meta-analysis have already examined the effect of ET on VO_2peak_ performance and reported the positive effects of ET on aerobic performance in healthy and unhealthy individuals [12,13,39]. Accordingly, our systematic review and meta-analysis showed a large effect of ET on VO_2peak_ performance. Previously published meta-analyses found a mean ES of 0.65 standard deviation units, representing an improvement in oxygen consumption of 22.8%, with length of training, pre-training VO_2peak_, and duration of training bouts accounting for 59% of the total variation in delta VO_2peak_ [36]. For instance, Huang et al. [40] pooled together 41 RCTs including 2102 older individuals aged 60 years and older. The standardized ES showed a higher moderate effect of 0.64 ± 0.05 (95%CI 0.56–0.73), representing a net increase in VO_2peak_ of 3.78 ± 0.28 mL/kg/min (95%CI 3.24–4.33) or a 16.3% improvement, compared with control groups. Of note, the adaptations in the pulmonary, cardio-vascular and neuromuscular systems that improve the delivery of oxygen from the atmospheric air to the mitochondria and enhance the control of metabolism within the muscle cells may explain the improvement in VO_2peak_ following ET [41]. More specifically, up to 66% of the improvement of VO_2peak_ performance in males was due to augmented maximal cardiac output, while the increase in VO_2peak_ in older females was mediated by a wider arteriovenous oxygen content difference [42].

### 4.2. Participants Characteristics

In the aforementioned systematic reviews and meta-analyses, both genders and healthy and unhealthy individuals were merged in the same experimental group [12,13]. For instance, the present systematic review and meta-analysis reported that ET induced larger effect on VO_2peak_ performance in healthy subjects than unhealthy individuals. Contradictory results have been reported for young adults, in which the VO_2peak_ increase after ET was greater in the girls (+9.1%) than in the boys (+4.6%) [43]. The results of the previous study may be explained by the lower hemoglobin concentrations and higher levels of body fat in females than males [44,45,46].

### 4.3. Dose–response Relationships of Endurance Training on VO_2peak_

Our analyses revealed that none of the training prescription variables predicted ET effect on VO_2peak_ performance in healthy and unhealthy older individuals. In contrast to our meta-regression data, a previous meta-analysis reported a larger effect of long-term continuous ET on VO_2peak_ performance than an ET of lower duration in young individuals [47]. Furthermore, in a previous meta-analysis, Huang et al. [16] reviewed 41 RCT and non-RCT studies that studied the effect of ET on VO_2peak_ in healthy sedentary older adults. They reported that only training intensity predicted ET on VO_2peak_. More specifically, training intensity at 66–73% heart rate reserve (HRR) elicited largest improvement in VO_2peak_ than 35–50%, 57–65%, and 75–80% of HRR In this sense, Huang et al. [40] reported that training length more than 20 weeks and training intensity of approximately 60% but less than 70% of VO_2peak_ were more effective for improving VO_2peak_ in older individuals aged 60 years and over.

Only one study examined the effect of training volume on VO_2peak_ performance in healthy older individuals [27]. The authors reported a slightly greater effect of longer training duration than shorter duration without significant difference between them. In fact, the American College of Sports Medicine (ACSM) suggested that older participants may need longer periods of time to progress and adapt to ET [48]. Furthermore, according to our findings, Coker et al. [31,32] reported that both moderate and high intensity ET similarly improved VO_2pea_k over 12 weeks in unhealthy individuals.

However, according to training prescription variables, the expert opinion in the ACSM recommended to undertake moderate-intensity continuous exercises for a minimum of 30 min, on 5 days each week, or 20 min of vigorous exercises 3 days each week, or a combination of the two in addition to the activities of daily living [49]. Finally, the optimal dose–response for improving VO_2peak_ performance in healthy and unhealthy individuals aged 70 years and older is still unknown. Further studies are urgently needed to determine the effects of ET on VO_2peak_ performance according to training prescription variables.

### 4.4. Limitations

Our study has some limitations that warrant discussion. First, we tried to identify effective dose–response relationships. However, findings from the univariate analyses have to be interpreted with caution because such analysis does not enable to control/adjust for other training prescription variables. Second, we showed moderate to high heterogeneity between the included studies which could have affected our study outcomes. Third, we did not control our quantitative synthesis for variables such as the type of test that predicted VO_2peak_ performance, which may have influenced training induced adaptations. Another limitation is given by the small number of studies included in the present systematic review and meta-analysis. Furthermore, our systematic review and meta-analysis is not registered in PROSPERO. However, this limitation may be considered disputable since much of the information required by PROSPERO is the same as what is recommended when completely reporting a protocol using the PRISMA-P checklist. Finally, the included studies used different methods to estimate VO_2peak_, which make it difficult to compare between them.

## 5. Conclusions

The present systematic review and meta-analysis showed that ET is an effective method for improving VO_2peak_ performance in healthy and unhealthy older individuals. Sub-group analyses revealed that healthy individuals showed greater gains in VO_2peak_ performance compared with unhealthy individuals. The meta-regression showed that none of the training prescription variables predicted ET effect on aerobic performance. Future studies are needed to elucidate relevant ET-related variables that allow the predictive analysis of dose–response relationships following ET in older adults.

## Figures and Tables

**Table 1:** Effect of endurance training on VO_2peak_ considering different moderating variables (health status, gender, training and session duration, and training frequency).

Independent Variables	Number of Studies	Number of Effect Size	SMD Estimate	SE	95% CI	*p*	Heterogeneity, Q Statistic (*p* Value)	F Value and (*p*) between Groups
	Main effect							
Pooled effect (REML model)	12	18	2.60	0.79	0.97to 4.31	0.004	127.810 (<0.001)	NA
	Health status							
Healthy	6	9	2.25	0.51	1.09 to 3.04	<0.001	74.91 (<0.001)	F_1,15_ = 13.52 (<0.001)
Unhealthy	5	8	1.53	0.53	0.41 to 2.65	0.011		
Both (Healthy and Unhealthy)	1	1	15.00	2.54	9.62 to 20.37	<0.001		
	Gender							
Both (Female and Male)	7	10	1.38	0.91	−0.54 to 3.30	0.148	91.94 (<0.001)	F_1,15_ = 3.17 (0.07)
Female	2	2	3.63	1.66	0.11 to 7.14	0.044	102.03 (<0.001)	F_1,15_ = 0.46 (0.63)
Male	3	6	2.23	1.3	−0.31 to 4.71	0.088	119.08 (<0.001)	F_1,15_ = 2.41 (0.12)
	Training duration							
<13 weeks	4	9	2.26	0.97	0.22 to 4.23	0.032	115.37 (<0.001)	F_1,16_ = 0.54 (0.47)
≥13 weeks	8	9	2.87	0.84	1.05 to 4.58	0.004		
	Session duration							
20–30 min	2	2	1.32	2.13	−3.19 to 5.84	0.544	77.17 (<0.001)	F_1,15_ = 1.01 (0.37)
31–45 min	6	11	3.58	1.05	1.27 to 5.76	0.005		
46–65 min	5	5	2.25	1.13	−0.14 to 4.64	0.064		
	Training frequency							
3 per week	9	13	2.89	1.01	0.71 to 5.00	0.012	123.67 (<0.001)	F_1,16_ = 0.11 (0.73)
4–5 per week	3	5	2.19	1.74	−1.48 to 5.853	0.225		

CI, confidence interval; df, degrees of freedom; NA, not applicable; SD, standard deviation; SE, standard error; SMD, standardized mean differences.

## Data Availability

All data were already included in the main text of the manuscript.
